# The Metabolic Profile of Tumor and Virally Infected Cells Shapes Their Microenvironment Counteracting T Cell Immunity

**DOI:** 10.3389/fimmu.2019.02309

**Published:** 2019-10-04

**Authors:** Isabelle Magalhaes, Ohad Yogev, Jonas Mattsson, Anna Schurich

**Affiliations:** ^1^Department of Oncology-Pathology, Karolinska Institutet, Stockholm, Sweden; ^2^Division of Infection and Immunity, University College London, London, United Kingdom; ^3^Gloria and Seymour Epstein Chair in Cell Therapy and Transplantation, Division of Medical Oncology and Hematology, Princess Margaret Cancer Centre, Toronto, ON, Canada; ^4^Department of Medicine, University of Toronto, Toronto, ON, Canada; ^5^Department of Infectious Diseases, King's College London, London, United Kingdom

**Keywords:** metabolism, tumor, T cell, virus, hypoxia

## Abstract

Upon activation naïve T cells undergo metabolic changes to support the differentiation into subsets of effector or regulatory cells, and enable subsequent metabolic adaptations to form memory. Interfering with these metabolic alterations leads to abrogation or reprogramming of T cell differentiation, demonstrating the importance of these pathways in T cell development. It has long been appreciated that the conversion of a healthy cell to a cancerous cell is accompanied by metabolic changes, which support uncontrolled proliferation. Especially in solid tumors these metabolic changes significantly influence the tumor microenvironment (TME) and affect tumor infiltrating immune cells. The TME is often hypoxic and nutrient depleted, additionally tumor cells produce co-inhibitory signals, together suppressing the immune response. Interestingly, viruses can stimulate a metabolism akin to that seen in tumor cells in their host cells and even in neighboring cells (e.g., via transfer of virally modified extracellular vesicles). Thus, viruses create their own niche which favors viral persistence and propagation, while again keeping the immune response at bay. In this review we will focus on the mechanisms employed by tumor cells and viruses influencing T cell metabolic regulation and the impact they have on shaping T cell fate.

## Introduction

In recent years the fundamental importance of energy regulation in immune cells has been appreciated and has created the research field of “Immunometabolism.” Since there are excellent current reviews discussing the metabolic regulation of T cells in detail (1–3), we will here only give a short overview and then focus on the role of the tumor or virally infected target cell in manipulating T cell fate. The reader might also find Table 1 helpful, summarizing the main mechanisms discussed for a quick overview. Naïve T cells are relatively quiescent cells that have a low energetic demand. They predominantly make use of mitochondrial oxidative phosphorylation (OXPHOS). Upon antigen encounter and activation by professional antigen presenting cells, T cells increase the expression of nutrient transporters, especially glucose transporters 1 and 3 (GLUT1 and 3) (26) and start utilizing glycolysis even in the presence of sufficient oxygen. Glycolysis provides fast energy and biological building blocks required for cell division and effector function. Alongside, mitochondrial biogenesis is also activated and mitochondrial mass increased, allowing for increased respiration. Furthermore, a recent study highlights the important role of distinct mitochondrial metabolic pathways in regulating T cell proliferation and effector differentiation (27). Mitochondrial function is critical, and its disruption seems to be an underlying mechanism of T cell exhaustion (28–30). Upon the resolution of an acute insult (such as an acute viral infection) a proportion of the effector T cell pool differentiates into memory cells. This conversion is accompanied by the cell's metabolism refocussing on OXPHOS and fatty acid metabolism, while reducing glycolysis. Memory T cells have an increased mitochondrial mass and contain mitochondria with densely packed cristae linked to more efficient OXPHOS (31), poised to mount a fast recall response. In the situation where the immune response fails to deal with the insult effectively and T cells are subjected to persisting antigenic challenge like in cancer and chronic viral infection, effector responses become less vigorous and instead of developing into classical memory cells, formation is skewed toward exhaustion. Here we will discuss aspects of how the metabolic profile and signaling by target cells shapes their microenvironment and the impacts on T cell function, differentiation and fate decision.

**Table 1:** Table summarizing metabolic pathways and their role in tumors/TME and viral infection.

**Mechanism**	**Tumor**	**Virus**
Glycolysis	(i) Glucose depleted TME (ii) Inhibition of effector T cells (4, 5)	Stimulation of increased glycolysis in host cell for viral production and in neighboring cells via exosomes e.g., HTLV, HIV, KSHV, EBV (6, 7)
Hypoxia/pseudohypoxia	Stabilization of HIF1-α: (i) Enhances glycolysis and acidosis of TME (8) (ii) Expression of ectoenzymes CD39/CD73 increasing extracellular adenosine levels (9) (iii) Upregulation of PD-L1 (10)	Stabilization of HIF1-α mimicking the effect of hypoxia (termed: pseudohypoxia) e.g., KSHV, EBV, HCV, HCMV, HPV (11, 12)
Lactate production	(i) Acidification of TME (ii) Inhibition of effector T cells (13, 14) (iii) Inhibition of CD4+ Th1 T cells (15) (iv) Promotion of Treg (15) (v) Induction of regulatory macrophages (16) (vi) Upregulation of PD-L1 (17)	Lactate secretion from (i) infected cells and (ii) neighboring cells stimulated by virally infected cells, creates microenvironment supporting viral propagation (e.g., KSHV)? (4, 18)
Amino acid depletion	(i) Increased glutaminolysis leads to glutamine depletion in TME (19) (ii) Expression of indoleamine 2,3-dioxygenase leads to depletion of tryptophan (20) (iii) Recruitment/induction of MDSC, which can deplete the essential amino acids cysteine and arginine	Recruitment of MDSC e.g., to HBV infected liver (21) Inhibition of amino acid uptake mediated by HIV Vpu protein (22)
	Inhibition of effector T cells	
	Induction of Treg (23)	
Lipid metabolism	Induction of increased release of fatty acids by adipocytes to fuel tumor (24)	Induction/modulation of fatty acid production e.g., CMV, KSHV, HCV, Zika, Dengue (25)

## The Increased Glycolysis in Cancer Cells Impacts on T Cells

Reprogramming of energy metabolism has been recognized as one of the hallmarks of cancer cells (32). It is well established that cancer cells have increased glucose uptake, which is fermented to lactate even in the presence of oxygen, a process known as aerobic glycolysis (or Warburg effect). Of note, glucose can also be further metabolized through the mitochondrial tricarbolyxic acid (TCA) cycle in tumors (33).

Glucose is an important source of carbon for the production of amino acids, nucleotides, and fatty acids. Oncogenic mutations of the phosphatase and tensin homolog (PTEN) and the phosphoinositide 3-kinase (PI3K) pathway lead to the reprogramming of glucose metabolism and increased glucose uptake via stimulation of glucose transporters in cancer cells [recently reviewed by Marbaniang et al. (34)]. Increased transcription of glycolysis genes in KRAS mutated colorectal cancer cell lines (35) is another example of the impact of oncogenic mutations on the cellular metabolic state. Mutations in the tumor suppressor gene p53 was shown to play a role in glucose metabolism, and interestingly also in mitochondrial activity and lipid metabolism (36).

The sustained consumption of glucose by tumor cells eventually leads to a decrease of glucose levels in the TME. Competition for glucose between tumor and T cells has been shown to decrease IFN-γ production by CD8+ T cells and to limit T cell antitumor functions (4, 5). Conversion of glycolysis intermediates by the pentose phosphate pathway generates NADPH (that can serve as an electron acceptor), which is needed for tumor cells to scavenge reactive oxygen species (ROS) and maintain redox homeostasis. ROS produced by tumor cells participate in the oxidative stress T cells encounter in the TME, and interestingly, Tregs are more resistant than conventional CD4+ T cells to oxidative stress-induced cell death (37).

## The Influence of Hypoxia on T cell Function

**Figure 1:**
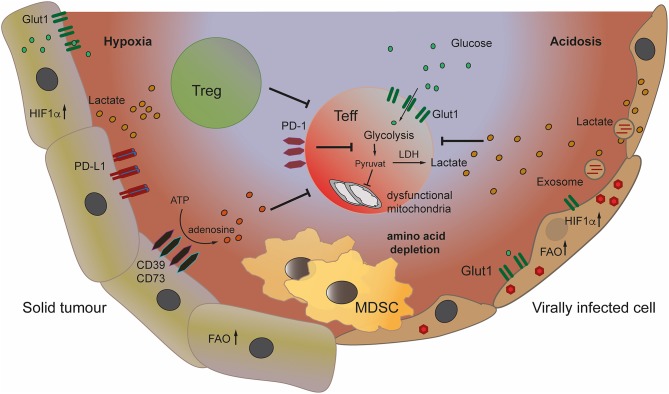
Metabolic changes in tumor and virally infected cells can create a suppressive microenvironment leading to inhibition of effector T cells. The architecture of the tumor microenvironment (TME) can create hypoxic areas, leading to stabilization of HIF1-α in tumor cells and increase of glycolysis, depleting the TME of glucose. Increased glycolysis and production of lactate leads to acidification of the TME. Lactate inhibits effector T cells, while promoting Treg. Tumor cells convert ATP to immune suppressive adenosine by expressing the ectoenzymes CD39/CD73. The recruitment and induction of myeloid derived suppressor cells (MDSC) to the TME and to virally infected organs increases immune inhibition. Viruses also induce glycolysis and lactate production in infected and neighboring cells through the transfer of viral signaling molecules to their target cells via exosomes.

Reduced blood flow in some tumor areas results in low oxygen levels (hypoxia) and acidification as discussed below and shown in Figure 1. Hypoxia leads to the stabilization of the transcription factor hypoxia-inducible factor 1-α (HIF1-α). HIF1-α in cancer cells promotes glucose uptake by upregulation of GLUT1, GLUT3, and increased expression of glycolytic enzymes further promoting glycolysis and acidosis (8). In an elegant study Walton et al. have demonstrated that high acidity in the context of hypoxia results in inhibition of mTOR signaling in T cells and induction of T cell anergy. mTOR is a key sensor of nutrients and a major regulator of cellular metabolism and effector T cell function (38). Earlier studies had demonstrated that mTOR inhibition in the presence of TCR triggering drives T cell anergy (39), while promoting Treg development (40). Taken together these mechanisms might conceivably play a role in the tumor microenvironment *in vivo*, with both T effector cell inhibition and increased Treg numbers detrimental to tumor control. Various viruses (e.g., HCV and human papillomaviruses) have also been shown to manipulate the host cell's metabolism by promoting through different mechanisms HIF1-α stability and activity (11, 12) in the absence of hypoxia leading to subsequent increased glycolysis. In a recent report, influenza A (H1N1) virus led to proteasome inhibition and in turn stabilization of HIF1-α in normoxic conditions, however the impact on viral replication remains to be determined (41).

Apart from driving increased glycolysis HIF1-α induces expression of the ectoenzyme CD73 (9) and the expression by cancer cells and Tregs of the tandem ectoenzymes CD39/CD73 which generate extracellular adenosine from the degradation of extracellular ATP. The binding of adenosine to the 2A2-adenosine receptor (A2AR) expressed by many immune cells including T cells, inhibits anti-tumor T cells (42). Finally, HIF1-α is also involved in the upregulation of PD-L1 by tumor cells (10). Interestingly, a deregulated oxidative metabolism in tumors and the associated hypoxia in the TME, correlated with resistance to PD-1 treatment (43).

A recent publication shows that hypoxia and glucose deprivation lead to down-regulation of MHC class I molecules on tumor cells facilitating immune escape. This finding was accompanied by tumor cells losing their sensitivity to IFN-γ mediated induction of MHC upregulation (44). Consequently, tumor cells evade killing by activated IFN-γ producing T cells creating another hurdle for T cell therapies.

## TUMOR Cells Produce Lactate That Promotes Immune Suppression

In addition, the lactate secreted by glycolytic tumor cells into the TME adversely impacts on effector T cells. Lactate inhibits CD8+ T cell proliferation, cytokine (IL-2 and IFN-γ) production, and cytotoxicity (13, 14), but also induces T cell apoptosis (45). These effects on activated CD8+ T cells by increased lactate levels are mediated through inhibition of NFAT upregulation and inhibition of phosphorylation of p38 MAP kinase and JNK (14, 45). A recent report demonstrated that exogenous lactate can reduce the frequency of Th1 CD4+ T cells and their IFN-γ production, while increasing the expression of FoxP3 and the frequency of regulatory CD4+ T cells (Tregs) (15). Expression of cell surface GLUT1 is lower in Tregs as compared to other CD4+ T cell subsets, and Tregs appear not to rely on glycolysis but rather lipid oxidation and OXPHOS (46). FoxP3 was shown to play a role in inducing resistance to the suppressive effects of lactate by mediating distinct metabolic adaptation in Tregs (18). The accumulation of lactate in the TME leads to TME acidification known to decrease T cell proliferative capacity and inducing anergy (47) (Figure 1). Interestingly, expression of the lactate receptor GPR81 by macrophages was shown to play a role in inducing immune regulatory genes and regulating inflammation (16).

Lactate was also shown to upregulate programmed cell death ligand-1 (PD-L1) expression in lung cancer cells (17). Interestingly, PD-L1 blockade on tumor cells inhibited their mTOR kinase activity and decreased the expression of glycolytic enzymes (4), which might contribute to the success of anti-PD-L1 checkpoint blockade.

Finally, the increased concentration of lactate in the TME seems to be advantageous to some tumors, as lactate can be converted to pyruvate to fuel the TCA cycle. Glycolytic tumor cells can therefore produce the fuel for neighboring cells in heterogenous tumors (48), while at the same time suppressing the immune response.

## TUMOR Cells do not Solely Rely on Glucose as a Source of Energy but UTILIZE Amino Acids Leaving the TME Depleted

TCA activity can be replenished by mitochondrial metabolism of various amino acids such as alanine, cysteine, leucine, and fatty acids (33). Recent data shows that at least some tumors depend on the non-essential amino acid glutamine (in humans, the most abundant amino acid in the circulation), as a source of nitrogen needed for nucleotide biosynthesis, and of carbon to fuel the TCA cycle (49). Metanalysis of studies assessing the metabolic profile in patients with cancer have revealed that in addition to the expected increase in lactate in tumor tissues, other metabolic changes can be identified (50, 51). Glutamate and kynurenine were the two most frequently elevated metabolites when 343 pairs of tumor/normal samples were compared.

Glutamine metabolism is upregulated by many oncogenic mutations (52). The *MYC* oncogene is one of the most frequently amplified genes in human tumors. MYC upregulates glutamine transporters, and MYC-transformed cells are dependent on glutamine metabolism (19). This can lead to reduced levels of glutamine in the TME, compared to normal tissues, resulting in limited availability for T cells. In order to sustain the energetic demands of cell proliferation and differentiation following T cells activation, T cells upregulate glutamine uptake, and enzymes for glutamine metabolism. Interestingly, extracellular glutamine deprivation and subsequent decreased intracellular pool of the glutamine-derived α-ketoglutarate promotes a shift in murine CD4+ T cells toward Treg differentiation (53). This was supported by data using human T cells where inhibition of glutaminolysis (conversion of glutamine into TCA cycle metabolites) promoted Treg differentiation (54).

Competition for glutamine may therefore represent an additional mechanism of immunosuppression in the TME.

Furthermore, many tumors constitutively express indoleamine 2,3-dioxygenase (IDO) which catabolizes the essential amino acid tryptophan depleting it from the TME inhibiting T cell proliferation (20). Depletion of tryptophan suppresses CD8+ effector T cell proliferation but again promotes Treg differentiation via activation of the GCN2 kinase (55). Tryptophan metabolism also releases the immunosuppressive catabolite kynurenine that activates the aryl hydrocarbon receptor which also promotes Treg differentiation (56). IDO inhibitors have been tested in clinical trials, but responses were overall disappointing either as single agents, or in combination with anti-PD1 therapy leading to a halt of some combination therapy phase III trials (57).

Tumors are well known to induce and attract myeloid derived suppressor cells (MDSC), which crucially can suppress both innate and adaptive immune responses (Figure 1). One mechanism being nutrient depletion by the sequestration of cysteine and the production of arginase-1, an enzyme leading to the break down of arginine, both amino acids being essential for T cells. In contrast to other cells T cells cannot convert the oxidized precursor cystine to the reduced amino acid cysteine and are dependent on extracellular levels (23). The depletion of arginine which has been demonstrated to contribute to suppression of T cell responses in cancer (58) is also operative in chronic viral infection. The HIV protein Vpu antagonizes amino acid uptake into CD4+ T cells (22), while in chronic hepatitis B virus (HBV) increased numbers of MDSC found in the infected liver correlate with low levels of arginine (21). As a consequence of the above combined mechanisms T cells in the TME and in chronic viral infections can find themselves depleted of essential amino acids, leaving them little ability to function effectively.

## The Role of Lipid Metabolism in the Regulation of T Cell Responses

An enhanced lipid metabolism is crucially required for the synthesis of cell membranes in blasting and proliferating T cells (59) and highly organized lipid rafts in the membrane of effector T cells which enable the organization of the immunological synapse (12). A perturbation of the cholesterol and fatty acid homeostasis leads to a reduction in effector T cells. Furthermore, the development of T cell memory has been shown to be dependent on increased mitochondrial fatty acid oxidation (60, 61). Like proliferating T cells, proliferating cancer cells require fatty acids for the synthesis of membranes and other molecules. Many tumor cells acquire fatty acids through *de novo* synthesis, however some ovarian, prostate and breast cancers rely on the uptake of exogenous fatty acids (62). Tumor cells have been shown to communicate with adipocytes to enhance provision of fatty acids (24), establishing a link between obesity and increased risk of cancer. Viruses likewise manipulate their host cell's lipid metabolism, for example human cytomegalovirus (CMV) induces an increase in fatty acid production to synthesize lipids for incorporation into the viral envelope (63). Hepatocytes infected by Hepatitis C virus (HCV) are forced to increase lipogenesis and gluconeogenesis to support viral particle production via sophisticated mechanisms involving viral proteins and interference with host miRNAs (64, 65). Since hepatocytes are vital in regulating systemic glucose and lipid homeostasis these manipulations by HCV lead to a significantly increased risk for patients to develop metabolic disorders. Pathogenic Flaviviruses, such as Zika and Dengue virus also rely on their host's lipid metabolism to complete their life cycle and thus interfere with this pathway to remodel intracellular membranes to allow virion biogenesis (25).

The importance of lipid metabolism in both cancer and viral infection make these pathways interesting candidates for therapeutic intervention.

## TUMORS, TME and T Cells are Heterogenous

One should keep in mind that tumor cells are proliferating, rapidly evolving cells, and metabolic changes are very heterogenous across different cancers, between patients with the same type of cancer, and within the same patient since spatial and temporal tumor cell heterogeneity also occur (66). In addition to intrinsic tumor cell differences, the heterogeneity of the tumor microenvironment (including oxygen levels/perfusion levels) influences the metabolic changes in tumor cells, which in turn as well shows heterogeneity. Tumor metabolomic analysis provides important information, however more efforts for instance in standardization of sampling, data analysis, choice of sample (cell lines, tissues, or blood), to ensure that only cancer specific changes are detected, are needed. These caveats are also true for the study of many chronic viral infections, especially where culture models are hard to establish and/or *in vivo* studies are limited to humanized models or primates where humans are the exclusive natural host of a virus as in HIV, HBV, HCV, and EBV.

As discussed above, metabolic suppression in the TME is important in inhibiting effector T cells and many solid tumors are devoid of much T cell infiltrate. Since T cell memory formation is equally dependent on metabolic programs, this can also be inhibited and skewed toward T cell deletion or dysfunction. Indeed, T cell exhaustion or Treg development occur in the TME instead of classical memory differentiation. It could be argued that exhaustion is a distinct type of memory, since exhausted T cells can be long lived, do retain limited effector functions and exert control over persistent viral infections (67–69). Thus, anti-viral CD8+ T cells with exhausted phenotype(PD-1 intermediate/high, low IFN-γ/effector cytokine production) can for example maintain a low viral load in patients with untreated chronic HBV (70). Furthermore exhausted T cells can be reinvigorated by stimulation with cytokines such as IL-12 (71) and are targeted in immune checkpoint inhibitor therapy (69).

## Viruses Manipulate the Metabolism of Both Their Host Cell and Cells in Their Microenvironment

Viruses acquire both the energy and the building blocks needed to synthesize progeny virions from their host. For this reason, it is not surprising that many viruses manipulate their host cell metabolic pathways and associated signaling cascades [reviewed in (6, 7)]. Interestingly, many of these metabolic changes mimic those found in cancer. This suggests that in the case of oncogenic viruses, these metabolic alterations also contribute to cellular transformation. An example of shared metabolic alteration between viruses and cancer is the induction of the Warburg effect. Similar to many cancer types, different viruses were shown to shift glucose metabolism and redirect the glycolysis end product, pyruvate, away from mitochondrial OXPHOS. Interestingly different viruses developed diverse mechanisms to manipulate glucose metabolism in their host cells. Moreover, increased glycolysis and reduced OXPHOS were shown to support both viral replication and latency, by activating biosynthetic pathways supporting viral propagation.

In the last decade, there is accumulating evidence that viruses not only manipulate the infected cells but also communicate and manipulate other cells in their microenvironment. One method viruses use for this is manipulation of extracellular vesicle (EV) secretion from the host cell (Figure 1). An increasing number of viruses has been shown to manipulate EV-secretion and cargo(72–84).

The field of EV has been extensively studied in the last years, mainly in cancer. Tumor-derived EVs were shown to have a dramatic effect on tumor growth and metastasis [reviewed in (85–87)]. Interestingly, it was shown that both cancer cells and viruses use EV to alter the metabolism of cells in their microenvironment. A fascinating example of this phenomenon comes from the two oncogenic gammaherpesviruses Epstein Barr Virus (EBV) and Kaposi's sarcoma herpesvirus (KSHV). Both viruses establish latency quickly after primary infection. Though during latency, these viruses express only a small subset of their genome, both viruses were shown to have a complex effect on their host metabolism (88). KSHV was shown to shift glucose metabolism from mitochondrial OXPHOS to aerobic glycolysis and to induce fatty acid synthesis and glutaminolysis (89–94). One of the driving forces for these metabolic alterations is the virally encoded microRNAs, which are thought to downregulate different genes which are involved in the regulation of OXPHOS and by that shift cells to more glycolytic metabolism (94). Importantly it was shown that these microRNAs are also transferred from infected cells to non-infected cells in the microenvironment using EVs (95–97) and that the viral microRNAs are active in these cells to induce similar metabolic phenotype (97) Similar to KSHV, EBV was also shown to alter its host cell metabolism (98). Specifically, the latent protein LMP1 was shown to shift host cell metabolism from OXPHOS to aerobic glycolysis (98, 99) by inducing expression of multiple genes, such as GLUT1. In EBV-induced carcinomas this increased glycolysis promotes MDSC expansion (100) leading to tumor immunosuppression as discussed. Additionally, LMP1, which is expressed in around 30% of EBV-driven Hodgkin Lymphomas, is also involved in stimulating regulatory T cell responses (101). LMP1 can be transferred in EV secreted from infected cells and thus manipulate EV-recipient cells (2, 102, 103). This suggests that similarly to KSHV, EBV can use EV to manipulate neighboring cells and thereby modulate its microenvironment.

Why do viruses alter their host's metabolism? One clear advantage is the activation of biosynthetic pathways to support viral replication. Redirecting pyruvate away from mitochondria and reducing OXPHOS can free different carbon molecules for the synthesis of nucleotides, amino acids and lipids or for protein glycosylation. Permissiveness of CD4+ T cells to HIV has been shown to be strongly influenced by the metabolic activation status of the T cells. CD4+ T cells with high rates of OXPHOS and glutaminolysis where the most susceptible (104, 105). Indeed, HIV infection could be significantly reduced by blocking glutaminolysis (105). In the case of latent viruses, the advantage of altering their host cell's metabolism is less obvious. Since during latency, there is a minimal expression of viral proteins, these viruses are completely dependent on cellular replication to maintain and replicate their genome. Adopting a “cancer-like” metabolism might support uncontrolled cell division, which results in maintenance and amplification of the viral genome.

Altering the metabolic state of cells in the microenvironment might suggest other advantages for infected cells. For example, in KSHV infection, it was shown that altering the metabolism of non-infected cells leads to the secretion of high-energy metabolites. These metabolites are being taken up by infected cells supporting their growth. Therefore, it is suggested that viruses can use EVs to create a specific niche which supports infected host cell growth (97).

Moreover, altering the metabolic phenotype of the niche could also allow viruses to escape the immune system. Since, T cells as part of their differentiation and activation need to undergo dramatic reprogramming of their cellular metabolism (3, 106) a low glucose high lactate microenvironment restricts T cells, dampening their effector function (4, 18). This raises the intriguing hypothesis that by manipulating their host cell's metabolism viruses attenuate T cell function by creating a suppressive microenvironment.

## Outlook and Therapeutic Implications

A better understanding of tumor metabolism is obviously important in order to target tumor cells as well as to counteract their immunosuppressive impact on anti-tumor T cell responses. As described below different strategies are therefore being explored. One approach consists in the intratumor delivery by nanoparticles of RNA interference that silences lactate dehydrogenase A (LDHA) (107). The observation in preclinical models that the effect of anti-PD1 treatment in a model of melanoma, was improved in mice with LDH-A deficient tumors (108), and that, the deletion of LDHA in myeloid cells was shown to induce T cell antitumor immunity against lung carcinoma (109), further validates the targeting of LDHA. Expression of catalase by chimeric antigen receptor (CAR) T cells improved the protection of CAR T cells against oxidative stress induced in part by ROS in the TME (110). A recent report showed that acetate could be used as an alternative carbone source and rescue the functions (e.g., IFN-γ production) of exhausted tumor infiltrating T cells, and glucose-restricted CD8+ T cells (111).

Autophagy is a catabolic process that allows cell survival and maintenance of cell metabolism in face of stressful conditions such as nutrient starvation. In tumor cells, autophagy appears to play different roles by promoting tumor suppression but also tumor initiation (112). A better understanding of autophagy in tumors could therefore potentially be exploited to develop novel anticancer treatments. Note that in the context of viral infections, autophagy can again play a dual role by promoting or limiting viral replication (113).

Despite viruses amending the immune response, as discussed above, anti-viral responses often successfully eliminate the infecting agents or keep them life-long under control as evidenced by the rare occurrence of CMV or EBV-mediated disease in healthy individuals despite up to 90% of the human population being persistently infected (12). This has led to the interesting idea of repurposing anti-viral T cells against (114). In a recent study Rosato et al. demonstrated that anti-viral T cells can target tumors when these were loaded with exogenous viral peptide. This strategy was made even more efficient when combined with check-point blockade (114) potentially opening up new therapeutic avenues. It remains to be determined if such a strategy impacts on anti-viral control. An important question will be whether utilizing anti-viral T cells could over time lead to their exhaustion and/or reprogramming into Treg in the suppressive TME. Thus, the choice of viral peptide targets and combination with other strategies will be critical; especially considering that a persistent common virus such as EBV is oncogenic if uncontrolled (115).

Altering cell metabolism is one of the hallmarks of cancer. However, it is becoming clear that this effect is not limited to the tumor cells, and as part of tumor development, the metabolic phenotype of the TME is also dramatically changed. Additionally, viruses can mimic this phenotype, affecting the metabolism of both their host cells and cells in their microenvironment. Despite considerable advances, we still have some way to go in understanding how these metabolic alterations affect T cell response and how they could successfully be used to target cancer and chronic viral infection. However, it is clear that the metabolic profiling of antigen specific T cells and their target cells should now be part of the development of new therapeutic strategies.

## Author Contributions

All authors have contributed to writing the article, and have read the manuscript.

### Conflict of Interest

The authors declare that the research was conducted in the absence of any commercial or financial relationships that could be construed as a potential conflict of interest.
